# An Update on the Clinical Management of HIV and Tuberculosis Co-Infection in Pregnancy: TB Preventative Therapy, Long-Acting ARVs, and Bedaquiline-Based Regimens

**DOI:** 10.1007/s11904-025-00746-z

**Published:** 2025-06-16

**Authors:** Tim Crocker-Buque, Michael A. Moso, Justin T. Denholm, Kasha P. Singh

**Affiliations:** 1 Department of Infectious Diseases, Peter Doherty Institute of Infection and Immunity, The University of Melbourne, Melbourne, VIC Australia; 2 Victorian Infectious Diseases Service, The Royal Melbourne Hospital, Melbourne, Australia; 3 Victorian Tuberculosis Program, The Royal Melbourne Hospital, Melbourne, Australia

**Keywords:** Tuberculosis, HIV, Pregnancy, Treatment, Prevention

## Abstract

**Purpose:**

This update addresses HIV/TB co-infection management in pregnancy, focusing on new treatment options.

**Recent Findings:**

Pregnancy with HIV increases TB risk and worsens treatment outcomes. While long-acting antiretroviral therapies (LA-ART) like cabotegravir/rilpivirine and lenacapavir exist, data on their safety and efficacy in pregnant individuals are limited. Treating both HIV and TB is crucial, but pregnancy’s physiological changes complicate drug management. Standard ART and TB preventive therapy (TPT) with isoniazid are recommended after excluding active TB, despite some concerns about adverse outcomes when combined with ARV treatment. For active drug-resistant TB, the new 6-month BPaLM regimen (bedaquiline, pretomanid, linezolid, moxifloxacin) is not recommended in pregnancy due to limited safety data on pretomanid. Instead, a 9-month regimen is preferred, though bedaquiline and pretomanid are likely safe. More research on these new therapies in pregnant populations is needed.

**Summary:**

While standard ART remains the recommended approach for HIV/TB co-infection in pregnancy, further research is crucial to establish the safety and efficacy of newer LA-ART and bedaquiline-based TB regimens in this high-risk population. Concerns around the safety of TPT in pregnancy remain unanswered and further prospective research is urgently needed.

## Introduction

Both human immunodeficiency virus (HIV) infection and pregnancy are associated with increased risk of tuberculosis infection (TBI, also known as latent tuberculosis infection), progression of TBI to active disease and poorer outcomes with treatment. TB is the leading cause of morbidity and mortality amongst persons living with HIV (PLWH) worldwide, who account for around 6–7% of TB cases, but over 10% of TB deaths [1, 2]. Progression to TB disease is over 10 times more likely in people with untreated HIV than for those without HIV infection [3], and increases with lower CD4 + T cell count [4, 5]. Even with effective antiretroviral therapy (ART), the risk of TB disease is higher than amongst the general population [1, 6].

An increased risk of developing TB disease is also independently associated with pregnancy [2, 7, 8]. A recent systematic review and meta-analysis reported that the relative risk of TB progression is 1.3–1.4 in pregnancy and rises higher to 1.9-2.0 in the postpartum period, increasing with advancing maternal age [8]. In this context, the 2024 Global Tuberculosis Report calls for scaling up of TB prevention programs for pregnant women [9]. TB contributes up to 15% of all maternal mortality globally, highlighting the importance of optimal management of HIV/TB coinfection in pregnant women [9]. In a recent observational study from South Africa using programmatic data to look at TB in pregnancy, outcomes were generally poor. Risk factors included older age, HIV infection, poorer TB treatment outcome and late presentation to antenatal services [10].

The magnitude of risk for TB disease during pregnancy and post-partum in PLWH is not well characterised and is likely to alter with CD4 + T cell count and use of ART. A meta-analysis from 2020 examining the association between pregnant PLWH and TB disease only identified 5 relevant studies (all prior to 2014), reporting a 1.56 times risk of TB disease amongst pregnant PLWH compared to those without HIV (RR 2.56, 95% CI = 1.57–4.17) [11]. Amongst pregnant women living with HIV, concurrent TB infection is associated with poor maternal and infant outcomes with increased maternal mortality, increased risk of pregnancy-related complications and poorer birth outcomes, including neonatal TB infection and adverse effects on the family [12–18].

Despite the significant burden of TB amongst PLWH during pregnancy and post-partum, alongside considerable complexities in management including the impact on drug levels and drug-drug interactions, there is a paucity of solid evidence to help guide complex clinical decision-making. Here we provide an update on the implications for clinical management of HIV and TB co-infection in pregnancy, with a focus on the evolving evidence of the use of TB preventive therapy (TPT) for treatment of TBI, the use of long-acting ART, and the use of bedaquiline-based regimens for rifampicin resistant (RR) and multi-drug resistant (MDR) TB infection.

## An Update on the Management Of HIV in Pregnancy

The preferred therapy for pregnant people with HIV is dolutegravir (DTG) and two nucleoside reverse transcriptase inhibitors (NRTIs), either tenofovir disoproxil fumarate (TDF)/tenofovir alafenamide (TAF) plus emtricitabine (FTC)/lamivudine (3TC); or abacavir (ABC) plus 3TC in those who are HLA-B*5701 negative and negative for hepatitis B surface antigen (HBsAg). These recommendations are based on the U.S. perinatal HIV guidelines (last update December 2024) [19], which are broadly aligned with preferred therapies listed in the 2024 European AIDS Clinical Society guidelines [20], and the British HIV Association draft guidelines on management of HIV in pregnancy and the postpartum period 2024 [21]. Individuals may commonly be receiving alternate ART regimens prior to pregnancy, including the fixed-dose combination (FDC) bictegravir (BIC)/TAF/FTC or two-drug FDC, DTG/3TC, which are listed as first-line therapies for HIV in non-pregnant adults. The 2024 update to the US perinatal HIV guidelines recently changed the BIC recommendation to ‘alternative therapy’ from ‘insufficient data to recommend use in pregnancy’ [19], based on safety data from the Antiretroviral Pregnancy Registry demonstrating no increased risk in overall birth defects from 539 reported pregnancies with first trimester BIC exposure [22]. Recommendations have also been updated to advise continuation of BIC/TAF/FTC for people who become pregnant on this regimen and have been stable with a suppressed viral load [19, 23]. Alternative regimens are recommended for people receiving cabotegravir (CAB) as pre-exposure prophylaxis (PrEP), which are considered in Sect. 2.1 below.

Data for use of two-drug DTG/3TC regimen in pregnancy are limited; however, both component drugs are listed as preferred regimens in pregnancy as part of a three-drug ART regimen and have favourable pharmacokinetic (PK) profile in pregnancy. Clearance of 3TC has been shown to increase by 22% in pregnancy compared to non-pregnant individuals, although overall 3TC exposure levels were comparable to reported levels in non-pregnant individuals [24]. DTG exposure has been shown to decrease compared to postpartum in the second and third trimesters of pregnancy by 37% and 29% respectively; however, trough DTG levels remained more than ten-times above the in vitro 90% inhibitory concentration for wild-type HIV [25]. No dose adjustment is indicated for either drug. DTG/3TC is currently not recommended for initiation of ART in ART-naïve pregnant people. For those already on DTG/3TC with a suppressed viral load prior to pregnancy, the option to continue DTG/3TC with more frequent viral load monitoring can be discussed as part of shared decision-making. Two small observational studies have demonstrated ability of DTG/3TC to achieve and maintain virologic suppression in pregnancy, with undetectable viral load achieved before delivery in 20 individuals in an open-label pilot study in Brazil [26], and in 10 of 11 in a retrospective cohort in Italy (in the one non-suppressed individual, viral load was 53 copies/mL) [27]. There were no cases of vertical transmission in either study.

### Long-Acting ARVs

Two long-acting ART regimens have been approved by the US Food and Drug Administration (FDA) for treatment of HIV, cabotegravir (CAB)/rilpivirine (RPV) and lenacapavir (LEN). However, currently, their availability remains limited in most settings. Long-acting (LA) CAB/RPV (CAB/RPV LA) is approved for use in non-pregnant individuals to replace a stable regimen when plasma viral load has been suppressed, administered as a monthly or bimonthly intramuscular injection. CAB/RPV LA has also been used successfully in viraemic individuals with adherence challenges who are unable to achieve viral suppression despite intensive efforts to support adherence to oral ART [28, 29]. LEN is currently approved for use in multi-drug resistant HIV when added to a regimen of optimized background ART and is administered as a 6-monthly subcutaneous injection.

There are limited data on the safety and effectiveness of CAB/RPV or LEN for treatment of HIV in pregnancy. As of 31st January 2024, only 20 cases were reported to the Antiretroviral Pregnancy Registry for CAB use in first trimester and one case for LEN [22]. RPV is non-teratogenic, but reduced drug exposures have been observed in pregnancy of up to 50% for oral RPV [30]. A report of one individual receiving bimonthly CAB/RPV LA during pregnancy demonstrated comparable CAB concentrations at 35 weeks’ gestation compared to non-pregnant individuals, but 70–75% lower RPV concentration, although virologic suppression was maintained and vertical HIV transmission did not occur [31]. PK modelling has predicted RPV trough concentrations would fall below 4-times the protein binding-adjusted effective concentration 90 (EC90) for 97% and 45% of pregnant people in third trimester receiving bimonthly or monthly CAB/RPV LA, respectively [32]. In contrast, CAB trough concentrations were predicted to be above 4-times the EC90 in close to 100% of individuals receiving either monthly or bimonthly injections.

Both LEN and CAB/RPV LA are effective as PrEP and may have advantages over daily drugs based on improved adherence, however availability is even more limited than for TDF/FTC tablets [33]. A PK sub-study recently evaluated long-acting CAB for HIV PrEP in 55 pregnant women as part of the HPTN 084 open label extension study. Median CAB trough concentrations were observed to decrease by 20% in third trimester compared to pre-pregnancy levels, however the concentrations remained above the protocol-defined exposure targets [34]. Pregnancy outcomes were similar in individuals who received CAB during pregnancy compared to oral TDF/FTC PrEP [35].

Guidelines from the National Institutes of Health (NIH; USA) do not recommend starting CAB based regimens in pregnant people and declare insufficient data to make recommendations on its use in those trying to conceive. In the case where someone fully suppressed on a CAB based regimen becomes pregnant, it is recommended that the CAB-based regimen is continued with frequent viral load monitoring (or consider switching due to insufficient data) [19]. For those who are considering switching regimens prior to conception to prevent fetal exposure, CAB/RPV injections must be stopped at least one year before conception to ensure that these long-acting drugs are fully eliminated. For people who become pregnant while receiving PrEP, including drugs not yet approved for PrEP during pregnancy such as LA injectable agents, registration with the Antiretroviral Pregnancy Registry is encouraged [19]. In people with a history of CAB exposure for PrEP, ritonavir-boosted darunavir (DRV/r)-based regimens are preferred for initial ART due to concerns about integrase inhibitor resistance mutations [36]. In other situations, DRV/r is now recommended as an alternative, rather than preferred, for use in pregnancy (see Sect. 2.2).

Interim data on LEN use in pregnancy were reported as part of the PURPOSE 1 study, which evaluated LEN for HIV PrEP in cisgender women [37]. 5,368 women were randomised. 193 pregnancies occurred in the LEN group, of which 105 (54.4%) were complete at the time of interim data analysis. Pregnancy outcomes were reported to be similar to those expected for the population. Spontaneous abortion was reported in 10.4% (20/193) in the LEN group, compared to 15.5% (34/219) in the TAF/FTC and 12.2% (12/98) in the TDF/FTC arm. There were no documented HIV acquisitions on LEN. Amongst pregnant participants HIV infection occurred in no participants in the LEN group, compared with 4 participants in the F/TAF group and 1 participant in the F/TDF group. Additional data, including LEN PK in pregnancy are awaited.

Regimens including LEN may pose challenges for treatment in those who develop TB whilst on these regimens, including pregnant women. Significant decline in levels of LEN is expected during therapy with rifampicin (Table 1). Alternative regimens for TB treatment may need to be considered, or reinforcement of ART with additional agents, although this may not be possible.

### Protease Inhibitors in Pregnancy

In most cases, protease inhibitors (PI) are now recommended as alternative rather than preferred ARV for use in pregnancy and in people trying to conceive. Treatment-experienced individuals or those with other concerns for integrase-resistant virus may require initiation or continuation of PIs in pregnancy. This includes pregnant people with a history of CAB-LA exposure for PrEP, in whom PIs are preferred for initial ART regimens due to concerns about integrase inhibitor resistance mutations. The preferred PI in pregnancy is darunavir, boosted with low-dose ritonavir (DRV/r) [19]. Both DRV and ritonavir have not been associated with teratogenicity [22]. Twice-daily dosing of DRV/r is recommended in pregnancy, given lower DRV exposure has been observed in third trimester pregnancy with DRV/r 800 mg/100 mg daily dosing (39% reduction in DRV exposure) compared to 600 mg/100 mg twice-daily dosing (26% reduction) [38]. Boosting with ritonavir is preferred over cobicistat, due to significant loss in cobicistat pharmaco-enhancing effect during pregnancy, with 50% and 49% reduction in DRV and cobicistat exposure observed in third trimester respectively, compared to postpartum [39]. Ritonavir-boosted atazanavir (ATV/r) is also an option with the advantage of daily dosing.

## Management of TB with HIV Co-Infection in Pregnancy

In PLWH, treatment of both HIV and TBI is recommended. During pregnancy, complexities arise due to physiological changes that may alter drug distribution and levels, and the possibility of drug therapy influencing pregnancy outcomes. Initial licensing or registration studies excluded pregnant individuals or individuals who may become pregnant and often also PLWH, and pharmacovigilance systems are variable, particularly in low/middle income countries (LMICs) where most PLWH and TB occurs [40]. There is thus a lack of reliable data regarding drug use in pregnancy and in PLWH, making recommendations difficult. This is particularly problematic where outcomes are poor such as in pregnant PLWH with TBI.

The physiological changes of pregnancy, including changes in fat and water distribution, higher plasma volume and cardiac output, as well as increased renal clearance and liver metabolism (through induction of various CYP enzyme systems), can have significant effects on TB therapeutic drug levels [41, 42]. Overall, the effect is reduced levels of many TB drugs, however dilution of maternal plasma proteins can also lead to decreased clearance of other drugs that are extensively protein bound [43]. Pregnancy-induced cholestasis can theoretically result in increased concentrations of drugs, such as rifampicin that are eliminated by hepatobiliary excretion [44]. Table 1 presents a summary of the PK/PD characteristics of drugs used to treat TB with an overview of considerations when prescribing in HIV co-infection and pregnancy.

Due to the complex nature of PK/PD effects of pregnancy and the lack of study data, doses of most drugs are not recommended to be altered. The few PK studies looking specifically at TB medications during pregnancy in women with HIV are reassuring and suggest maintenance of study drug dosing is justified. These are presented in detail with additional detailed data on drug handling in an earlier review on this topic [45].

In brief, most PK data on medications for TB in pregnant women with HIV undergoing treatment for TB is available from analysis of samples taken during the Tshepiso study [17]. This is a prospective cohort evaluating the effects of TB on maternal and infant outcomes in women with HIV infection. In a sub-study of the Tshepiso trial, drug levels were measured on samples taken during pregnancy (> 36 weeks gestation) and 7 weeks postpartum and PK modelling undertaken [46, 47]. Results suggested no significant effect on levels of rifampicin (R), isoniazid (H), pyrazinamide (Z) and ethambutol (E) concentrations in pregnant women with HIV on treatment for TB using standard dosing.

The pharmacokinetics of isoniazid and efavirenz (EFV) were examined in detail using samples from the IMPAACT1078 study looking at the timing of isoniazid preventive therapy (IPT) in pregnant women with HIV [48]. Pregnancy increased clearance of isoniazid and EFV by 26% and 15% respectively [49], levels unlikely to be clinically significant in most cases. However the effects were influenced by genotypes of NAT2 and CYP2B6, raising the possibility that augmentation of the changes of pregnancy which may be of clinical importance in some people. Availability of genotypic testing remains limited.

Additionally, a recent study was undertaken to examine the PK and safety of the 3 monthly weekly rifapentine (P) and isoniazid (3PH) regimen for TB prevention in pregnancy included 20 women with HIV who were taking EFV-based therapy. In this study pregnant women without HIV (*n* = 30) had 28% lower clearance of rifapentine during pregnancy than post-partum, resulting in higher levels. In pregnant women with HIV, clearance was 30% higher than in those without HIV (*p* < 0.001) and did not change significantly between pregnancy and post-partum. Isoniazid PKs did not differ with pregnancy, nor HIV [50]. There were no differences in either drug in either population between the 2nd and 3rd trimesters. The reason for the differences observed is not known, however may be related to ART effects or HIV-associated differences in absorption and levels remained within those associated with successful TB prevention.

Most drugs used to treat TB are considered safe in pregnancy, with the exception of ethionamide (and its pro-drug prothionamide) that have been shown to be teratogenic in animal models, and the injectable aminoglycosides (e.g. amikacin), which are well known to be nephrotoxic and neurotoxic with a particular effect on the auditory vestibular nerve. Although the other drugs are considered safe, many have significant side-effects or potential toxicities, which may be more severe in pregnancy than in non-pregnant patients, however reports are inconsistent [51–53]. This includes those that are known to be hepatotoxic, including the rifamycins, isoniazid, pyrazinamide and pretomanid. HIV similarly has been recognised as a risk factor for adverse effects of TB medications; however attribution of adverse effects is difficult with coadministration of ART [54, 55]. Some guidelines suggest monthly monitoring of liver biochemistry should be undertaken in these groups [56, 57], however this may not be feasible in all settings and should not preclude the use of these agents in treatment for TB [58].

Given the physiological changes in pregnancy and the uncertainty of dosing levels for several key drugs, therapeutic drug monitoring (TDM) can play an especially important role to ensure both treatment effectiveness and reduction in risk of toxicity [59]. In practice however this can be laborious and requires timely laboratory testing and interpretation, which may be unavailable in places where HIV/TB in pregnancy is common.

**Table 1:** The pharmacokinetics, dynamics, and safety of drugs used to treat tuberculosis in pregnancy and additional considerations in people living with HIV

Drug	Administration and standard dose#	PK	PD	Side effects*	Considerations in pregnancy	Foetal effects	Considerations in HIV infection^	References
Rifampicin/ Rifampin	450-600 mg OD PO	Rapid absorption, decreased by food. High bioavailability (70%). Half-life 3–4 h. High distribution into tissues, lower into blood, low in CSF. Liver metabolized, mainly excreted in bile and faeces.	Inhibition of bacterial DNA-dependent RNA polymerase Potent CYP3A inducer (can increase its own metabolism) P-glycoprotein inducer 80% bound serum protein	Hepatotoxic	Considered safe	Anaemia risk, but generally considered safe	Weight and malabsorption risk important for correct dosing. Associated with low serum levels, especially with low CD4. May benefit from higher dosing. Lopinavir/ritonavir lowers Rif levels. Rif reduces levels of many ARVs, including many integrase and protease inhibitors, NNRTIs (except efavirenz, and in some cases nevirapine), capsid inhibitor lenacapavir and TAF (but not TDF). Dolutegravir or raltegravir may be used with rifampicin but dosing often increased from daily to bd Efavirenz may be used with rifampicin without dose adjustments.	[60–67]
Rifabutin	300 mg OD PO	Low oral bioavailability, long elimination half-life (∼45 h). CYP3A4 substrate. Compared with rifampin is more lipid soluble with more extensive tissue distribution and larger volume of distribution	Inhibition of bacterial DNA-dependent RNA polymerase Weak inducer of CYP450 P-glycoprotein inducer metabolism 72–85% bound serum protein	Uveitis	Limited data	Limited data	More expensive than rifampicin and may be hard to get. Serum concentrations are decreased by efavirenz due to CYP3A4 induction and conversely are increased by protease inhibitors. Dose adjustments may be needed. Reduces TAF levels (p-glycoprotein substrate) (but not TDF). Cabotegravir, dolutegravir and raltegravir may be used with rifabutin. Rifabutin should be used instead of rifampin with PI-based ART	[68–73]
Rifapentine	Latent disease: -3 month regimen w isoniazid: 900 mg if > 50 kg once weekly -1 month regimen w isoniazid: 600 mg daily PO if > 45 kg Active disease: -4 month regimen with moxifloxacin: 1.2 g DAILY PO -6 month regimen: 600 mg twice/week PO	Slow absorption, increased by fats. High bioavailability with food (70%). Half-life 14–15 h High distribution into tissues. Liver metabolized, mainly excreted in bile and faeces	Inhibition of bacterial DNA-dependent RNA polymerase CYP3A inducer. > 90% bound serum protein	Hepatotoxic	Likely to be safe, but limited evidence	Limited data, but likely to be safe.	Limited evidence for the use of Rifapentine alongside HIV treatment, but may have a lesser drug lowering effect than Rifampicin. Clearance is higher in people with HIV on an efavirenz-based regimen who are pregnant, but does not require dose adjustment. Reduces levels of (and should not be used with) TAF, NNRTIs (except efavirenz), PIs/cobicistat. Daily dosing of rifapentine is not recommended with integrase inhibitors	[50, 60, 61, 66]
Isoniazid	300 mg OD PO	Absorption decreased by food. High bioavailability (> 90%). Half-life 2–5 h (slow acetylators), 0.5–1.5 h (fast). High distribution into tissues. Liver metabolized, NAT2 dependent (acetylation status important), then CYP system. Mainly excreted in urine.	Mycolic acid synthesis inhibitor, preventing cell wall formation. Low protein binding (< 10%)	Hepatotoxic (especially slow acetylators (NAT2) and rapid metabolizers (CYP)). Peripheral neuropathy	Considered safe No significant effects on drug exposure Distributed into placenta Needs close monitoring for toxicity.	Generally considered safe	Lower peak serum levels, irrespective of CD4 count. The drug regimen interactions that may cause the lower isoniazid concentration are not clear, but is more likely to be related to acetylator status (fast) and CYP2B6 system status.	[47, 49, 50, 66, 67, 74–78]
Ethambutol	15 mg/kg OD PO	Absorption decreased by food. High bioavailability (80%). Half-life 3–4 h Mainly renal elimination into urine.	Cell wall formation inhibitor 30% bound serum protein	Optic neuropathy	Considered safe Potentially higher clearance and this lower exposure.		Weight important for dosing. Associated with low serum levels (reduced bioavailability), irrespective of CD4.	[63, 64, 66, 67, 75, 79, 80]
Pyrazinamide	< 50 kg 1.5 g OD PO > 50 kg 2 g OD PO	Rapid absorption. Half-life 9–10 h Hepatic metabolism. Mainly excreted in urine.	Likely metabolic (fatty acid synthesis) disruption# Low protein binding (< 10%)	Hepatotoxic	Considered safe No significant effects on drug exposure with standard dosing.	Limited data on foetal effects	Low serum levels associated with CD4 count, with high risk of treatment failure.	[65–67, 76]
Moxifloxacin	400 mg OD PO or IV	Readily absorbed. Very high bioavailability (90%) Half-life 12–15 h Liver and renal metabolism, excreted in both urine and faeces.	DNA gyrase inhibitor 50% bound plasma protein	QT prolongation Tendinopathy Effects on mental health	Likely to be safe. Reduced drug exposure. Present in breastmilk.	Historically avoided due to concern for foetal cartilage development based on animal models. No evidence of increased foetal abnormalities in humans. Lower birthweight.	Reduced exposure with efavirenz.	[66, 81–86]
Levofloxacin	10-15 mg/kg OD PO or IV	Rapid absorption. Very high bioavailability (99%). Very high distribution into tissues. Excreted in urine, mainly unchanged.	DNA gyrase inhibitor 30% bound plasma protein				No anticipated drug-drug interactions and recommended ARVs in pregnancy	
Bedaquiline	400 mg OD PO (first 2 weeks) 200 mg thrice/week PO (> 2 weeks)	Readily absorbed, increased by fats. Bioavailability Very high distribution into tissues, present in CSF Very long half-life (4–5 months) Liver metabolized (CYP3A4), excreted in faeces.	Mycobacterial ATP synthase inhibitor > 99% plasma protein bound	QT prolongation	Reduced exposure at standard drug doses. Concentrates in breastmilk.	No evidence of harm, but very little research data. Possible lower birthweight.	Exposure increased with lopinavir/ ritonavir. Exposure reduced with efavirenz.	[66, 81, 85, 87–90]
Pretomanid / Delamanid	Preto: 200 mg OD PO Dela: 100 mg BD PO	Readily absorbed. Bioavailability about 50%, but increased by food. Half-life 15–18 h Liver metabolized, mix of urine and faeces excretion.	Cell wall inhibitor 80% plasma protein bound	Hepatotoxic QT prolongation Myelosuppression	Considered safe. Limited evidence, but good birth outcomes.	No evidence of teratogenicity (animal models, but limited human data)	No PK differences with dolutegravir-based regimens. Exposure reduced with efavirenz.	[66, 81, 85, 89, 91, 92]
Linezolid	600 mg OD PO or IV	Readily absorbed. 100% bioavailability. Half-life 5–7 h. Urinary excretion of drug and metabolites.	Ribosomal inhibitor 30% bound plasma protein	Myelosuppression Peripheral and optic neuropathy	Limited evidence. Postnatally highly variable drug concentrations at different stages of pregnancy. Higher toxicity risk. Present in breastmilk at low levels.	Myelosuppression No evidence of teratogenicity.	Caution with SSRI and TCA drugs	[66, 81, 85, 93–96]
Clofazimine	100-200 mg OD PO	Absorption around 50%, increased with food. Lipophilic and deposits in fat tissues Very long half-life (25 days)	Likely metabolic inhibitor and cell membrane disruption# High plasma protein binding	Skin discoloration Intestinal dysfunction	Potential for increased fat stores to reduce drug concentration. Present in breastmilk.	Crosses placenta, causing foetal skin discolouration. No evidence of teratogenicity.	No evidence of differences. Potential increased exposure with lopinavir/ritonavir	[66, 81, 85, 97, 98]
Ethionamide / Prothionamide	Pro: 15-20 mg/kg OD PO (max 1 g)	Complete absorption with 100% bioavailability. Half-life 2–3 h Extensive liver metabolism. Difficult to achieve required concentrations.	Cell wall inhibitor 30% bound plasma protein	Gastrointestinal intolerance, hyperemesis Hypothyroidism	Avoid: poorly tolerated, reduces thyroid function.	Neural tube defects (animal models)	No evidence of significant differences in PK. No anticipated drug-drug interactions and recommended ARVs in pregnancy	[66, 83, 85, 99, 100]
Cycloserine / Terizidone	Cyclo: 10-20 mg/kg OD PO Teriz: 10-20 mg/kg OD PO, in 2 divided doses	Slowly absorbed, but good bioavailability (> 70%). Reduced by high fat meals Half-life 10 h Renal excretion into urine. Increased clearance in smokers.	Cell wall inhibitor Nil protein binding	Neuropsychiatric effects	Limited evidence, but well tolerated in pregnancy Present in breastmilk	Limited evidence, but no known teratogenicity	No evidence of significant differences in PK. No anticipated drug-drug interactions and recommended ARVs in pregnancy	[81, 83, 85, 101–105]
Para-aminosalicylic acid	150 mg/kg/day PO in 2–4 divided doses	60% bioavailability. Good CSF penetration. Short half-life (1 h), requiring large daily doses Hepatic metabolism and rapid elimination	Folate inhibitor 50–70% plasma protein binding	Gastrointestinal intolerance Hypothyroidism Hepatotoxicity	Very limited evidence, but has been used safely in pregnancy	Very limited evidence, but no clear teratogenicity	PAS reduces tenofovir exposure. Efavirenz reduces PAS exposure.	[66, 83, 106–110]
Amikacin	15 mg/kg/day IV (max 1 g)	Intravenous administration only. Half-life 2–3 h Excreted unchanged via kidneys in urine.	Ribosomal inhibitor < 10% protein binding	Nephrotoxic Ototoxic	Avoid Crosses placenta	Damage to kidneys and cranial nerve 8	No anticipated drug-drug interactions and recommended ARVs in pregnancy Tenofovir may cause renal impairment, and combining it with other medications that can also affect the kidney such as amikacin may increase that risk [111].	[66, 85, 112–114]

#Other dosing regimens are available for use in specific circumstances (e.g. renal impairment). Please check local prescribing guidelines. *Drug specific side-effects. General side effects common to many medications (nausea, vomiting, diarrhoea, fatigue) have not been included. ^This column presents a summary of important drug-drug interactions that have been documented in the literature. If no interaction is listed, it is unlikely an interaction is widely recognised. However, this is not comprehensive and other drug-drug interactions are possible. Please consult local prescribing guidelines. PK: pharmacokinetics; PD: pharmacodynamics; HIV: human immunodeficiency virus; OD: once daily; PO: by mouth; TAF: tenofovir alafenamide; TDF: tenofovir disoproxil; h: hours; NNRTI: non-nucleoside reverse transcriptase inhibitor; CSF: cerebro-spinal fluid; IV: intravenous; SSRI: selective serotonin reuptake inhibitor; TCA: tricyclic antidepressants

### Diagnosis of Tuberculosis Infection in HIV and Pregnancy

Diagnosis of TBI is by a combination of immunologic testing for reactivity to TB antigens, and exclusion of active disease [115]. Most commonly, immunological assessment is performed via tuberculin skin testing (TST, a cutaneous antigen challenge) or interferon-gamma release assay (IGRA; an in vitro antigen stimulation test). Both tests are widely used in cohorts with HIV or pregnancy, and have been demonstrated to risk stratify for future disease risk in both low and high prevalence settings [116–118]. Studies also suggest that test concordance is similar in pregnant and non-pregnant women where both tests are performed.

### TB Preventative Therapy

Both HIV and pregnancy increase the risk of progression of TBI to active disease and are associated with worse outcomes. ART and TPT act independently to decrease the risk of TB disease in PLWH, both interventions are recommended once active TB disease has been excluded, including in people who are pregnant [6, 57, 119–122]. However good quality data are lacking and some the results of studies are mixed. During pregnancy, exclusion of active disease may be complex as symptoms may overlap with those attributed to pregnancy [123, 124]. The increased engagement with medical services that may occur during pregnancy emphasises importance of pregnancy as an opportunity for undertaking TPT in PLWH.

Best practice for the use of TPT in pregnancy is not clear, with limited prospective studies on safety and efficacy [125]. Additional complexities exist in PLWH who are pregnant with some reports of adverse pregnancy outcomes with IPT, and the potential for drug-drug interactions. In low prevalence settings, such as the USA, local guidelines recommend deferring treatment for TBI in PLWH until 3 months post-partum, unless there is a high risk of developing TB disease, such as recent contact with an active case [126]. Whether or not this includes PLWH is unclear. US HIV guidelines for management of opportunistic infections, make a similar suggestion to delay therapy for TBI until after delivery if effective ART is being received and there is no close household contact with TB or recent seroconversion [127]. However, deferral of TPT without clear justification may lead to increased risk of progression to TB disease, with significant impact on pregnant women and babies, and careful consideration is essential.

Recommendations to delay TPT during pregnancy in PLWH, despite well documented harms of active TB in pregnancy, may be related to reports of adverse pregnancy outcomes associated with IPT in two studies. This includes recent policy directives in South Africa to defer or discontinue IPT in PLWH, then reinitiate immediately post-partum [128]. A summary of studies evaluating TPT in pregnancy is presented in Table 2. In a randomised trial of IPT initiated during vs. deferred (12 weeks postpartum) in 956 women with HIV on ART, a greater number of adverse pregnancy outcomes were seen in those on IPT initiated during pregnancy [48]. Recently published analysis from the ‘Brief TB’ study, is consistent with these results [129]. However, a recent large retrospective registry based study from South Africa and earlier randomised controlled trial found reduced risk of adverse outcomes with IPT (Table 2). The retrospective registry-based (Kalk Registry) publication also confirmed a reduction in cases of active TB.

No prospective studies have specifically considered effectiveness of TPT in pregnancy. Review of pregnancies during one large randomised controlled trial of 9 months of daily isoniazid versus 3 months of weekly isoniazid and rifapentine found no treatment-associated adverse maternal or neonatal effects in 125 women [50]. These findings align with retrospective cohort data finding no significantly increased risk of serious adverse effects in pregnant women receiving isoniazid in both Sweden and the United States [51, 130]. Prospective PK studies of isoniazid and rifapentine have also suggested that dose-adjustment is not required in pregnancy [131].

#### TPT Drug Regimens

Most data on efficacy of TPT and most experience is with IPT. WHO guidelines recommend a 6-month course (6 H) whilst 9 months is recommended in PLWH in the USA (9 H) [57]. However alternative regimens are listed in US guidelines for use in pregnancy (4R and 3 h) [127], with a comment that there is insufficient safety data for rifapentine use in pregnancy. In WHO guidelines, 6 H is recommended in all settings, with the alternative of 3HP and, in low prevalence settings 3–4 h or 3-4R are listed as alternatives [57]. Whilst evidence for repeated courses of TPT is lacking, the impact of TB disease in PLWH on morbidity and mortality of both mother and foetus, in combination with the safety of TPT on detailed review has contributed to the recommendation for at least 36 months of IPT (continuous/ongoing) in high prevalence settings, including amongst pregnant women [132].

**Table 2:** Summarises published data on the use of tuberculosis preventative therapy in pregnancy and post-partum in people living with HIV

Abbreviated citation	Study design, setting and population	Arms	Composite pregnancy outcomes including (low birth weight, preterm delivery, spontaneous abortion, stillbirth, or congenital anomaly)	Other outcomes
Gupta et al., NEJM 2019 [48]	RCT Eight countries with high TB prevalence HIV-infected pregnant women (⩾18 years old, 14–34 weeks gestation and 99.8–100% on cART)	Immediate isoniazid, started at study entry (2nd /3rd trimesters)(‘pregnancy-IPT’) and continued for 28 weeks (*n* = 477) Vs Deferred isoniazid, started at 12 weeks postpartum and continued for 28 weeks (*n* = 479) (‘postpartum-IPT’)	There was a higher incidence in the pregnancy-IPT group than in the postpartum-IPT group of an event included in the composite adverse pregnancy outcome (stillbirth or spontaneous abortion, low birth weight in an infant, preterm delivery, or congenital anomalies in an infant) (23.6% vs. 17.0%; difference, 6.7% points; 95% CI, 0.8 to 11.9).	
Theron et al., CID 2021 [133]	As above (Gupta et al., 2019)	As above - Identification and adjustment for multiple confounders of adverse pregnancy outcomes	Confirmed this increased risk of second- and third-trimester IPT exposure on composite adverse outcomes and low birth weight in a follow-up analysis, which identified and adjusted for multiple confounders of adverse pregnancy outcomes	
Cherkos et al., EClin Med 2023 [134]	As above (Gupta et al., 2019)	As above - Longer term outcomes IPT during pregnancy was also associated with adverse longer term growth outcomes in infants (especially among male infants)	Infants in pregnancy-IPT had a 1.47-fold higher risk of becoming underweight by 12 weeks (aHR 1.47 [95% CI: 1.06, 2.03]) than infants in the postpartum-IPT; increased risk persisted to 48 weeks postpartum (aHR 1.34 [95% CI: 1.01, 1.78]).	In sex-stratified analyses, male infants in the pregnancy-IPT arm experienced an increased risk of low birth weight (LBW) (aRR 2.04 [95% CI: 1.16, 3.68), preterm birth (aRR 1.81 [95% CI: 1.04, 3.21]) and becoming underweight by 12 weeks (aHR 2.02 [95% CI: 1.29, 3.18]) and 48 weeks (aHR 1.82 [95% CI: 1.23, 2.69]).
Tiendrebeogo et al., 2020 [135]	Randomized, controlled trial (TEMPRANO ANRS 12136) to evaluate the benefits and risks of 6-month IPT (vs. no IPT) and immediate antiretroviral therapy (ART) (vs. deferred ART) in 2052 West African adults [120, 121]	85 women became pregnant, including 14 who had voluntary abortions and 2 who died or were lost to follow-up while pregnant. Analysis is for the 69 other women	Those who received IPT while pregnant were more likely to have adverse pregnancy outcomes than those who did not (15 of 23 women [65%] vs. 25 of 46 [54%]). All the women who received IPT during their pregnancy had exposure to IPT during the first trimester, with a median duration of exposure of 34 days (interquartile range, 26 to 77; range, 10 to 98).	
Kalk et al., CID, 2020 [136]	Retrospective cohort Routine electronic clinical information systems from public sector health facilities, Western Cape, South Africa HIV-infected women on or initiating cART during pregnancy (41.8% newly initiated on cART and the rest already on cART)	Isoniazid duration unknown, based on “prescription” in the electronic record (*n* = 10 715) Vs No treatment (*n* = 41 227)	Women who received IPT were less likely to experience poor pregnancy outcomes ([aOR], 0.83 [95% {CI}, 0.78–0.87]); Association strengthened with IPT started after the first trimester compared with none (aOR, 0.71 [95% CI, 0.65–0.79]) or with first-trimester exposure (aOR, 0.64 [95% CI, 0.55–0.75]).	IPT reduced risk of TB by ∼30% (aHR, 0.71 [95% CI, 0.63–0.81]; absolute risk difference, 1518/100 000 women). Effect modified by CD4 cell count with protection conferred if CD4 count was ≤ 350 cells/µL (aHR, 0.51 [95% CI, 0.41–0.63]) vs. 0.93 [95% CI, 0.76–1.13] for CD4 count > 350 cells/µL). IPT was not associated with maternal mortality in adjusted survival analyses (aHR, 0.75 [95% CI, 0.46–1.22]). There was an increased association with severe liver injury (aHR, 1.51 [95% CI, 1.18–1.93]), although the overall number of events was low (*n* = 127)
Gupta et al., CID 2024 [137]	Pre-specified secondary analysis among women who became pregnant during AIDS Clinical Trials Group Brief Rifapentine- Isoniazid Evaluation for TB Prevention trial (ACTG A5279 BRIEF-TB) [138].	Standard nine-month IPT versus a 1-month isoniazid plus rifapentine regimen (1HP) in adolescents and adults with HIV in TB endemic settings Comparison between participants with an index pregnancy during IPT (IPT-exposed) or after IPT completion (unexposed).	Composite adverse pregnancy outcome (any non-live birth) and individual adverse outcomes (preterm delivery before 37 weeks gestational age and low birth weight less than 2500 g) IPT was associated with the composite adverse outcome adjusting for covariates at enrollment (adjusted relative risk [aRR] 1.98; 95% confidence interval [CI] 1.15, 3.41), but effect was attenuated when adjusted for covariates at pregnancy outcome (aRR 1.47; 95% CI 0.84, 2.55); IPT was not associated with preterm delivery (relative risk [RR] 0.87; 95% CI 0.32–2.42) or low birth weight (RR 1.01; 95% CI 0.29, 3.56).	
Taylor et al., Infect Dis Obstet Gyecol, 2013 [139]	Double-blind, RCT giving participants 6 m isoniazid plus B6, following which either isoniazid or placebo for 30 months or longer	Most IPT-exposed pregnancies (102 of 103) had first-trimester exposure, including a number of pregnancies conceived during IPT (median IPT initiation 341 days [48 weeks] before pregnancy outcome). Only 37% of women were on ART;	Reduced risk of composite adverse outcome (spontaneous abortion, stillbirth, preterm delivery, low birth weight, neonatal death, or congenital abnormality) among HIV-infected women exposed to long-term IPT in Botswana (aOR 0.6; 95% CI, 0.3–1.1)	

RCT: randomised controlled trial; TB: tuberculosis; HIV: human immunodeficiency virus; IPT: isoniazid preventative therapy; LBW: low birth rate; cART combination antiretroviral therapy; aOR adjusted odds ratio; aHR adjusted hazard ratio; aRR adjusted relative risk; CI confidence interval

Options for regimens should also take into consideration ART and potential drug-drug interactions. In a recent study using samples from the IMPACT P1078 study investigators looked at pregnancy-induced and pharmacogenetic-associated changes in PK and drug-drug interactions between isoniazid and EFV in pregnant women [48]. A reduction in exposure of both medications was seen during pregnancy, but the main determinants of drug concentrations were genotypes *NAT2* (isoniazid) and *CYP2B6* (EFV) [49]. However, the reductions seen were unlikely to be of clinical significance.

Rifamycin-based therapy is more complex (see Table 1). Administration of rifapentine with the integrase inhibitors (INSTI) BIC, elvitegravir (EVG) and CAB is not recommended due to potential for significant decrease in INSTI levels (Table 1). Administration with DTG is also associated with decrease in levels, however may be co-administered with careful monitoring for virologic efficacy. Once weekly rifapentine (900 mg) as in the 3HP regimen is not recommended for those who usually require twice daily DTG (e.g. with some INSTI-associated resistance substitutions), however PK data supports the use of daily DTG with weekly rifapentine as part of a 3 month TPT course [140]. With once daily rifapentine (600 mg) such as in the 1HP regimen, administration of DTG 50 mg twice daily is recommended, and this should be continued for 14 days after 1HP completion before switching back to once-daily dosing [141]. This is to allow for enzyme induction to resolve. Raltegravir (RAL) levels may be increased with weekly rifapentine (900 mg) and should continue to be administered at RAL 400 mg twice daily with no dose adjustment needed. RAL should not be administered with daily rifapentines due to decreased levels of the INSTI.

Rifampicin’s property as a strong inducer of P450 enzymes means it can induce metabolism of co-administered medications including ARTs including most PIs, the NNRTIs nevirapine (NVP) and rilpivirine (RPV), INSTIs BIC, EVG and CAB. RAL may be used at a dose of 800 mg twice daily and close monitoring for virologic response. Twice daily dosing of DTG may be considered in those without suspected INSTI-associated resistance mutations given in combination with rifampicin for treatment of TB. A recent phase 2 double-blind, placebo-controlled trial (‘RADIANT-TB’) in patients with CD4 + T cells > 100 cells/microL found comparable virological suppression in participants randomised to a second 50 mg DTG dose or placebo (in combination with a daily single tablet regimen of DTG/TDF/3TC [69]. No emergent INSTI resistance was seen. EFV-based ART (EFV 600 mg) may also be used safely with rifampicin-based TB therapy without further dose increases. In many settings EFV based therapy has been replaced as first line treatment for HIV with DTG containing regimens, however globally many PLWH remain on EFV-based therapy.

Overall, available data suggests that existing WHO recommended regimens for TBT can be safely used in pregnancy where indicated. The increased risk of TB disease during pregnancy and in the post-partum period is significant, and pregnant women with HIV infection should not be denied effective preventive treatments recognised to benefit both women and their children [142]. Guidelines for TB screening and preventive treatment lack specific screening and TPT recommendations for pregnancy and breastfeeding in PLWH. More studies are needed to help inform guidelines. A focus on shorter regimens may be beneficial in reducing the duration of exposure and thus potential risk for adverse outcomes in TBT during pregnancy in PLWH.

### Drug Sensitive TB

The management of drug sensitive TB in pregnancy has remained the same for many years with good evidence for standard 2-months HRZE followed by 4-months of HR at standard doses [143]. This treatment regime has been well evaluated and is considered safe. The most common adverse events identified are related to hepatotoxicity and this should be carefully monitored, particularly if acetylator status is not known (in relation to Isoniazid metabolism, see Table 1) [67, 81, 85]. However, any risk from the treatment regimen is significantly lower than progression of TB disease while pregnant or in the post-partum period. Pyridoxine (vitamin B6) should be added to the treatment regimen to reduce the risk of isoniazid associated peripheral neuropathy. Pyrazinamide is recommended as part of initial therapy for drug sensitive TB in guidelines from the WHO and International Union Against TB and Lung Diseases. This continues to differ from guidelines by the US Centers for Disease Control and Prevention (CDC) in which pyrazinamide is not recommended during pregnancy in due to ‘its effect on the fetus being unknown’ [126]. Instead, the recommendation is to use a 9-month treatment regimen without pyrazinamide.

It is important to ensure adequate drug exposures during pregnancy, due to the physiological changes resulting in increased distribution and clearance. In a detailed PK study in 27 pregnant women taking HRZE containing regimens (mainly using fixed dose combination tablets), PK of rifampin and pyrazinamide were similar in pregnancy and also between 2nd, 3rd trimesters and the post-partum period [67]. However, there was evidence of reduced exposures to isoniazid and ethambutol, and broad intra-participant variability at different time points. Changes may be required to maintain adequate drug levels, and all participants in this study were HIV negative.

Unfortunately, the four-month HPMZ (2 months HPMZ + 2 months HPM) regimens are not recommended due to a lack of evidence in pregnancy [143]. It’s likely that rifapentine is safe to use, in line with what is known about the other rifamycin drugs [50]. Similarly, moxifloxacin is used in RR/MDR regimens in pregnancy, and, although trial evidence remains limited, is likely to be safe [81]. The main benefit of the 4HPMZ regimen is the shorter duration, however the drug exposures remain similar with the exception of ethambutol. In practice, ethambutol is broadly considered safe in pregnancy and is sometimes dropped early in the treatment course if the mycobacterial isolate is confirmed as isoniazid sensitive and the patient is tolerating pyrazinamide [144]. If a pregnant patient is not tolerating routine HRZ(E) therapy then they should be treated with an equivalent drug-resistant TB regimen that is recommended in pregnancy.

Rifampicin presents the most important consideration for TB treatment regimens and the dosing considerations discussed in Sect. 3.2 above are also relevant for rifampicin-based treatment regimens for TB disease (as well as for its use in TBI). Interactions with rifampicin may be important for those on second line or PI based treatment regimens for HIV in which case alterations may need to be considered. In settings in which BIC-based regimens are commonly used, ART will need to be altered for those undergoing treatment with rifampicin. LEN is also significantly metabolized by CYP enzymes that are induced by rifampicin, leading to significant decrease in levels with coadministration (estimated at 84%). This would make LEN contraindicated with rifampicin administration [127]. The need for rifampicin during therapy with long-acting medications for HIV may necessitate administration of additional ARVs to compensate for reduced drug levels. Additional adverse events that may potentially arise in such scenarios will need to be carefully considered.

### Drug Resistant TB

Multi-drug resistant (MDR) TB in pregnancy is high-risk for both mother and foetus. A recent meta-analysis found risk of maternal death to be 7.5% (95% CI 3.2–12.8%), foetal loss 10.6% (6.0-16.3), and 12.9% pre-term birth (0.0–38%) [145]. However, many of these pooled studies were completed before the recent revolution in the treatment of DR TB. This has involved the discovery and marketing of two new classes of anti-mycobacterial drugs – the diarylquinoline class ATP synthase inhibitor drug bedaquiline and the bicyclic nitroimidazole class drugs delamanid and pretomanid, which affect mycolic acid synthesis and likely interrupt mycobacterial respiration function [146, 147]. Alongside these new drugs, several existing drugs have been repurposed and combined into novel RR/MDR TB drug regimens, including moxifloxacin and linezolid.

In 2022, the WHO published updated guidance that recommended a short (6-month) treatment course with bedqauiline (B), pretomanid (Pa), linezolid (L) and moxifloxacin (M) (BPaLM) in patients with RR or MDR tuberculosis [148]. This was based on the positive clinical trial results from the ZeNix and TB PRACTECAL [149–151]. However, these guidelines stopped short of recommending the 6BPaLM regimen in pregnancy, specifically due to the lack of safety evidence for pretomanid and the lack of inclusion in pregnant people within these clinical trials. Due to the relatively recent development of pretomanid within a new drug class, the majority of the safety data come from animal models, without a clear teratogenic effect [92]. However, further evaluation is underway due to a signal for testicular toxicity in animal models, with unknown implications at human dosing levels.

Instead, the 9-month all oral regimen is recommended, once fluroquinolone resistance is excluded. This involves 6 drugs for 4–6 months (bedaquiline, levo/moxifloxacin, clofazimine, ethambutol, isoniazid and pyrazinamide), with linezolid for the first 2 months. Once sputum smears are clear after 4 months, the treatment continuation phase includes levo/moxifloxacin, clofazimine, ethambutol and pyrazinamide for 5 further months [148]. It’s important to note that the standard 9-month regimen in people who are not pregnant includes ethionamide, which is contra-indicated in pregnancy due to the teratogenic effects found in animal models.

Subgroup analysis of clinical trial data and associated systematic reviews suggest that treatment with the bedqauiline-based regimens generally results in good outcomes for mother and baby. A systematic review by Alene and colleagues (2022) demonstrated high levels of success with quinolone, bedaquiline and linezolid containing regimens [152]. Of note, around 90 (of 275) patients received ethionamide, despite the expected teratogenicity risk, without evidence of congenital malformations. Almost none received pretomanid or delamanid in these studies. However, since this review, data on pregnancy in several smaller cohorts has also been published. In 43 pregnant women who received linezolid (95%), bedaquiline (93%) and/or delamanid (33%) containing regimens for RR/MDR TB in the endTB and STEM-TB studies, 98% had good TB treatment outcomes, 81% live births, and no congenital malformations reported [153]. Similar results were found in the 16 pregnancies in the TB-PRACTECAL trial, of which 15 people received pretomanid and 1 delamanid [88]. Given the greater simplicity and shorter duration of the BPaLM regimen, providing no clear evidence emerges of teratogenic risk with pretomanid, hopefully this regimen will soon be supported in pregnancy. Delamanid is already recommended in some guidelines, but this has not been tested as part of a bedaquiline-based regimen [154].

Choice of ART in PLWH undergoing treatment for drug-resistant TB during pregnancy is challenging, but important. Co-infection with HIV in adults with MDR-TB is associated with increased mortality, with use of ART found to mitigate this risk. DTG in combination with an NRTI backbone may be the most compatible regimen including in pregnancy in which it is recommended as ‘preferred’ [19, 155]. In those initiating ART, DTG is associated with more rapid viral suppression, and has lower potential for drug-drug interactions. It does not interact with bedaquiline. This contrasts with EFV, with which decreased bedaquiline serum exposure may be seen [156, 157]. EFV may also decrease levels of pretomanid due to CYP3A induction [158]. Alternatively, EFV may be substituted with NVP, although twice daily dosing is required. NVP is safe in pregnancy, although increased hepatotoxicity has previously been suggested. Levels of bedaquiline may be increased when co-administrated with boosted PIs, with the potential for increased exposure related toxicity [159].

## Psychosocial Support

Access to effective psycho-social support should be an essential component of person-centred care [160]. The need for such services is heightened in relation to HIV, TB and pregnancy, given the risk of stigma associated with each condition in many contexts, and the potential vulnerability of people affected by them. HIV, TB and pregnancy are all independent risk factors for depression, and psychological screening and access to counselling services should be provided [161–163]. Provision of psycho-social supports in the context of healthcare is often framed in terms of adherence support. This is an important component of healthcare services, and can assist in ensuring completion of TB treatment, maintenance of HIV control and participation in maternal testing and monitoring programs throughout pregnancy. However, counselling and other psycho-social supports are also valuable in themselves as a part of holistic healthcare, and improve self-reported outcomes [164]. Provision of counselling services for partners can also assist in promoting long-term connections with treatment programs after pregnancy [165].

Good adherence to therapy is necessary for optimal disease-related outcomes, including prevention of maternal-foetal transmission. Tangible support tools, such as pill boxes and timers, can improve adherence to medication [166]. However, equally important are less tangible supports, including peer-to-peer programs and availability of instant-messaging applications to allow rapid identification of issues and feedback about concerns [167, 168]. It is important to recognise that pregnant women with TB who have been established on effective therapy present minimal risk of transmission. They should be provided with access to regular supports provided for pregnancy, including antenatal review with healthcare practitioners. Education and support should be provided for returning to a full range of social interactions and community engagement.

## Conclusions

The combined risk of HIV and pregnancy on TB is significant, and TB in pregnant PLWH leads to poor maternal and infant outcomes. There is a lack of sufficient research specifically targeting the management of HIV/TB co-infection in pregnancy.

DTG-based regimens are preferred for pregnant people with HIV. Data on DTG/3TC in pregnancy is limited, but reassuring. LA-ART options like CAB/RPV and LEN exist, data on their use in pregnancy is limited and current guidelines do not recommend initiating CAB-based regimens in pregnant people or those trying to conceive. Guidelines for TB screening and preventive treatment lack specific recommendations for pregnancy and breastfeeding PLWH. A focus on shorter regimens may be beneficial in reducing the duration of exposure and thus potential risk for adverse outcomes in TBT during pregnancy in PLWH. For treatment of DR TB, the new 6-month BPaLM regimen, while effective in non-pregnant individuals, is not yet recommended in pregnancy due to lack of safety data on pretomanid in this population.

The exclusion of pregnant people from clinical trials remains a cause for concern. As both the management of HIV and TB are revolutionised, excluding people who are pregnant or at risk of becoming pregnant effectively excludes them from the benefits of newer, easier to use treatment regimens.

## Key References

- Bekker L-G, Das M, Abdool Karim Q, Ahmed K, Batting J, Brumskine W, et al. Twice-Yearly Lenacapavir or Daily F/TAF for HIV Prevention in Cisgender Women. N Engl J Med 2024;391:1179–92.
-
  - First study demonstrating the effectiveness of twice annual Lenacapavir injections for HIV prevention, with no adverse outcomes noted in pregnant participants.
- Salazar-Austin N, Hoffmann J, Cohn S, Mashabela F, Waja Z, Lala S, et al. Poor Obstetric and Infant Outcomes in Human Immunodeficiency Virus-Infected Pregnant Women With Tuberculosis in South Africa: The Tshepiso Study. Clin Infect Dis 2018;66:921–9.
-
  - Evaluated the effects of TB on maternal and infant outcomes in women with HIV infection and contributed much PK/PD data relating to pregnancy.
- Gupta A, Montepiedra G, Aaron L, Theron G, McCarthy K, Bradford S, et al. Isoniazid Preventive Therapy in HIV-Infected Pregnant and Postpartum Women. N Engl J Med 2019;381:1333–46.
-
  - PK/PD data on isoniazid and efavirenz in pregnancy were derived from this study of IPT in persons living with HIV.
- Mathad JS, Savic R, Britto P, Jayachandran P, Wiesner L, Montepiedra G, et al. Pharmacokinetics and Safety of 3 Months of Weekly Rifapentine and Isoniazid for Tuberculosis Prevention in Pregnant Women. Clinical Infectious Diseases 2022;74:1604–13.
-
  - Evaluated rifapentine and isoniazid as TPT in pregnancy showing that this regimen was effective despite observed PK/PD differences.
- Conradie F, Bagdasaryan TR, Borisov S, Howell P, Mikiashvili L, Ngubane N, et al. Bedaquiline–Pretomanid–Linezolid Regimens for Drug-Resistant Tuberculosis. New England Journal of Medicine 2022;387:810–23.
-
  - Evaluating short, all oral, bedaquiline-based regimens for drug resistant tuberculosis.
- Nyang’wa B-T, Berry C, Kazounis E, Motta I, Parpieva N, Tigay Z, et al. A 24-Week, All-Oral regimen for Rifampin-Resistant tuberculosis. N Engl J Med. 2022;387:2331–43.
-
  - Demonstrated short, all oral, bedaquiline-based regimens were effective for drug resistant tuberculosis, with sub-study published on pregnancy.

## Data Availability

No datasets were generated or analysed during the current study.
